# Diagnostic limitations of routine low-coverage CNV-seq in detecting mosaic paternal uniparental disomy in Beckwith-Wiedemann syndrome: a case report

**DOI:** 10.3389/fmed.2026.1900996

**Published:** 2026-09-08

**Authors:** Mengmeng Li, Lei Luo, Jianan Jiao, Yaying Cheng

**Affiliations:** Pediatrics Department, Hebei General Hospital, Shijiazhuang, China

**Keywords:** Beckwith–Wiedemann syndrome, CNV-seq, ICR, MS-MLPA, PUPD

## Abstract

**Background:**

Beckwith-Wiedemann syndrome (BWS) is an overgrowth disorder primarily caused by imprinting defects at 11p15.5. Mosaic paternal uniparental disomy (pUPD) accounts for approximately 20% of cases but is challenging to detect prenatally using routine low-coverage CNV-seq, as this approach has limited sensitivity for copy-number-neutral events such as uniparental disomy, particularly when present in a mosaic state.

**Case presentation:**

We report a newborn presenting with macroglossia, giant omphalocele, and a birth weight of 4,200 g (>97th percentile). Critically, routine prenatal CNV-seq on amniotic fluid revealed only variants of uncertain significance and was reported as normal for aneuploidies and pathogenic copy number variants. Postnatal whole-exome sequencing identified a 28.2 Mb region of absence of heterozygosity (AOH) on chromosome 11p15.5-p14.1, suggestive of mosaicism. Methylation-specific multiplex ligation-dependent probe amplification (MS-MLPA) subsequently confirmed the diagnosis of BWS due to mosaic pUPD, demonstrating hypermethylation of ICR1 and hypomethylation of ICR2. The giant omphalocele was successfully repaired via primary closure at birth. The patient underwent partial glossectomy at 23 months of age. No evidence of embryonal tumors was detected during 2-year follow-up.

**Conclusion:**

This case demonstrates that a normal prenatal CNV-seq result does not exclude BWS, particularly when classic clinical features are present. MS-MLPA remains the gold standard for diagnosing pUPD-related BWS. Early recognition, multidisciplinary surgical management, and regular tumor surveillance contributed to a favorable outcome.

## Introduction

Beckwith-Wiedemann syndrome (BWS) is the most common epigenetic overgrowth disorder, with an estimated birth prevalence of 1 in 10,000–13,700 (1). It is characterized by a predisposition to childhood cancers and a wide spectrum of clinical features, including macroglossia, abdominal wall defects, and neonatal hypoglycemia (2). Over 80% of cases are caused by defects in the imprinted gene clusters at chromosome 11p15.5, which regulate fetal growth (3). These defects include hypomethylation of ICR2 mosaic paternal uniparental disomy (pUPD, ∼20%), and hypermethylation of ICR1 (4).

While the clinical diagnosis of BWS is often straightforward in classic presentations, the molecular diagnosis — particularly for mosaic pUPD — remains challenging. Routine prenatal screening methods such as chromosomal microarray (CNV-seq) or karyotyping frequently miss these defects because pUPD is not a copy number variation but rather a copy number-neutral event (absence of heterozygosity, AOH) or an epigenetic abnormality. This can lead to diagnostic delays and missed opportunities for early tumor surveillance.

Here, we present a case of BWS due to mosaic pUPD11 in a newborn with a normal prenatal CNV-seq result. We aim to emphasize the diagnostic value of postnatal methylation-specific multiplex ligation-dependent probe amplification (MS-MLPA) and the importance of maintaining a high index of suspicion based on clinical findings.

## Case presentation

A female infant was born at 37 + 4 weeks of gestation to a 38-year-old gravida 3, para 3 mother via cesarean section due to preeclampsia and a prenatally diagnosed giant omphalocele. The pregnancy was notable for a prenatal ultrasound at 15 + 6 weeks showing a hyperechoic mass at the umbilical cord insertion site, suggestive of an omphalocele. Amniocentesis was performed at 20 + 5 weeks, and CNV-seq results were reported as normal for aneuploidies and pathogenic copy number variants, identifying only variants of uncertain significance (2q13q13 partial deletion and xq26.3q26.3 partial duplication). The technical parameters of the CNV-seq assay were: single-end 35-bp reads, 18 million reads (∼0.1–0.2 × depth), ≥100 Kb resolution, and a ≥ 30% mosaicism reporting threshold; the routine clinical pipeline did not implement AOH detection analysis. The family opted to continue the pregnancy following multidisciplinary counseling.

**Figure 1:**
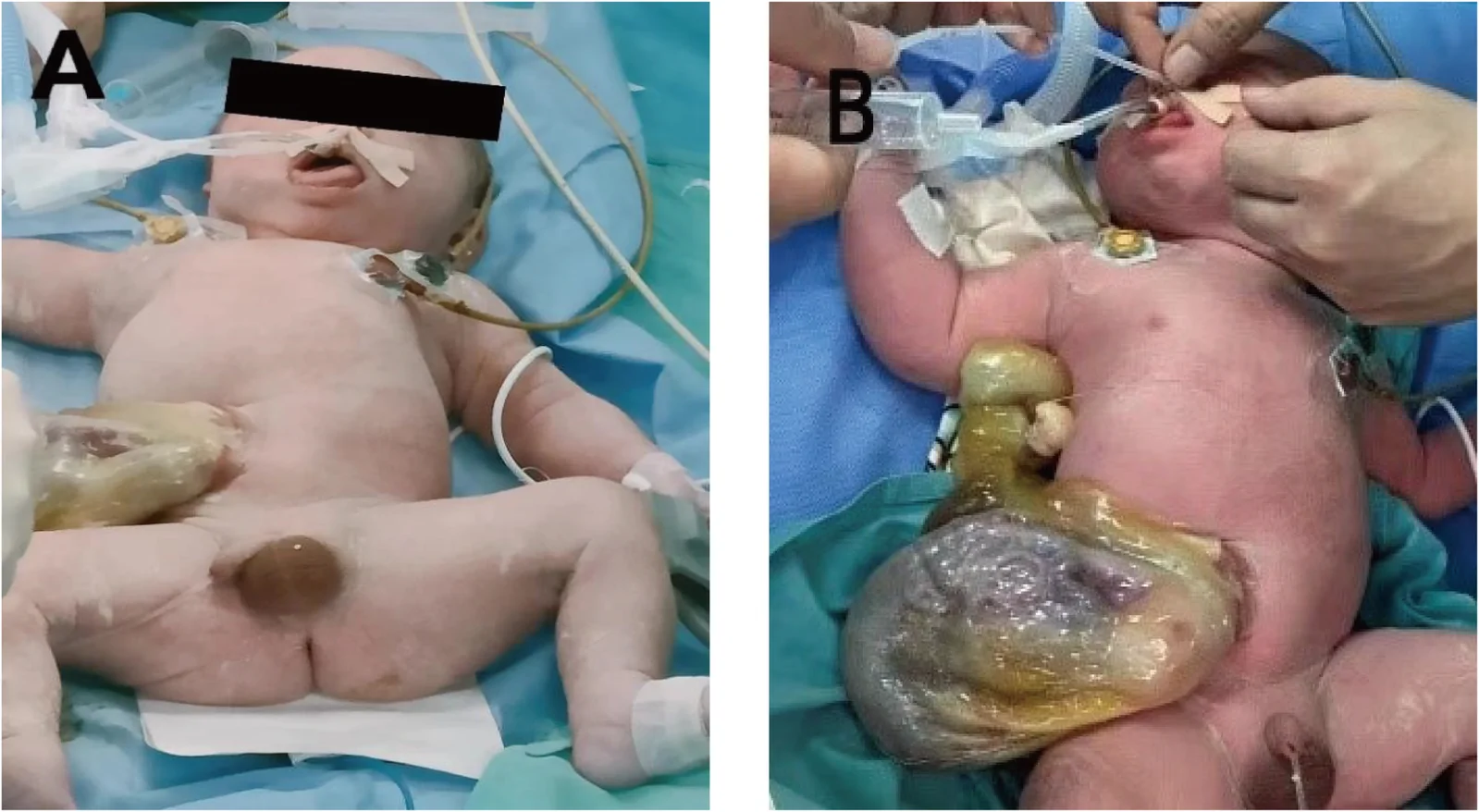
The condition of tongue and abdominal defects at the time of the child's admission. **(A)** the child's tongue protrudes out of the mouth; **(B)** shown the child's abdomen is protruding.

At birth, the infant weighed 4,200 g (>97th percentile). Physical examination revealed macroglossia with the tongue protruding from the mouth (Figure 1A) and a giant omphalocele with a 4 cm defect, containing most of the small intestine, colon, and part of the bladder (Figure 1B). Associated intestinal malrotation was confirmed during surgery.

Immediately after birth, the patient underwent a successful primary omphalocele repair combined with a Ladd procedure under general anesthesia. Postoperatively, she required mechanical ventilation for four days and parenteral nutrition, followed by gradual introduction of enteral feeds. Additional findings on postnatal evaluations included bilateral hydronephrosis (left renal pelvis 11 mm, right 13 mm), an atrial septal aneurysm, and a patent foramen ovale.

**Figure 2:**
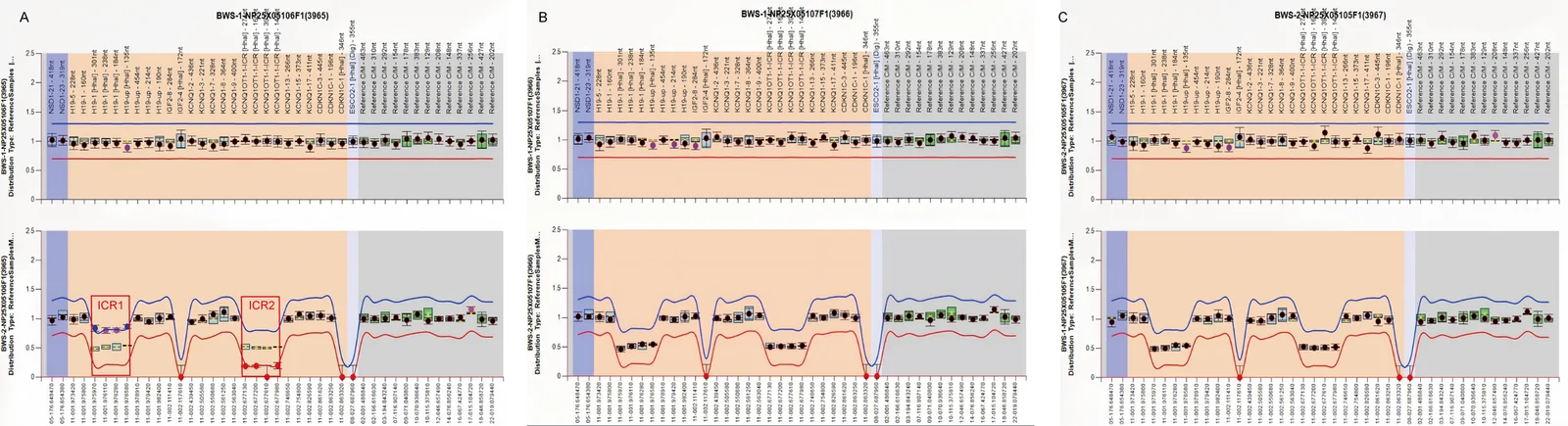
The MS-MLPA results of the child and his/her parents. **(A–C)** represent the MS-MLPA results of the child, the child's father, and the child's mother. The upper bar graph shows the changes in copy number, while the lower bar graph reflects the methylation changes in the ICR 1 and ICR 2 regions.

Given the clinical suspicion for BWS [score of 5 based on the 2018 international consensus criteria (5)], molecular testing was pursued. According to the 2018 International Consensus Criteria (5), cardinal features are assigned 2 points each and suggestive features 1 point each. Our patient presented with two cardinal features, macroglossia (2 points) and omphalocele (2 points), and one suggestive feature, birth weight greater than 2 SDS above the mean (1 point, 4200 g, >97th percentile), yielding a total clinical score of 5, which exceeds the threshold of 4 or more points for a clinical diagnosis of classic BWS. The prenatal CNV-seq had been performed due to the ultrasound finding of omphalocele in the absence of a clinical suspicion of BWS, as macroglossia and macrosomia were not apparent prenatally. It was only after birth, when the full clinical triad emerged, that targeted molecular testing for BWS was initiated according to the consensus guidelines (5). Postnatal whole-exome sequencing on peripheral blood revealed a 28.2 Mb region of absence of heterozygosity (AOH) at chromosome 11p15.5p14.1, suggesting possible uniparental disomy (UPD). Subsequent MS-MLPA analysis confirmed the diagnosis of BWS due to mosaic paternal uniparental disomy (pUPD), demonstrating hypermethylation at ICR1 and hypomethylation at ICR2 (Figure 2A). The parents' samples showed normal methylation patterns (Figures 2B,C). The specific degree of mosaicism was not quantifiable from the clinical MS-MLPA report, but the clear presence of both methylated and unmethylated alleles confirmed the diagnosis of mosaic pUPD.

**Figure 3:**
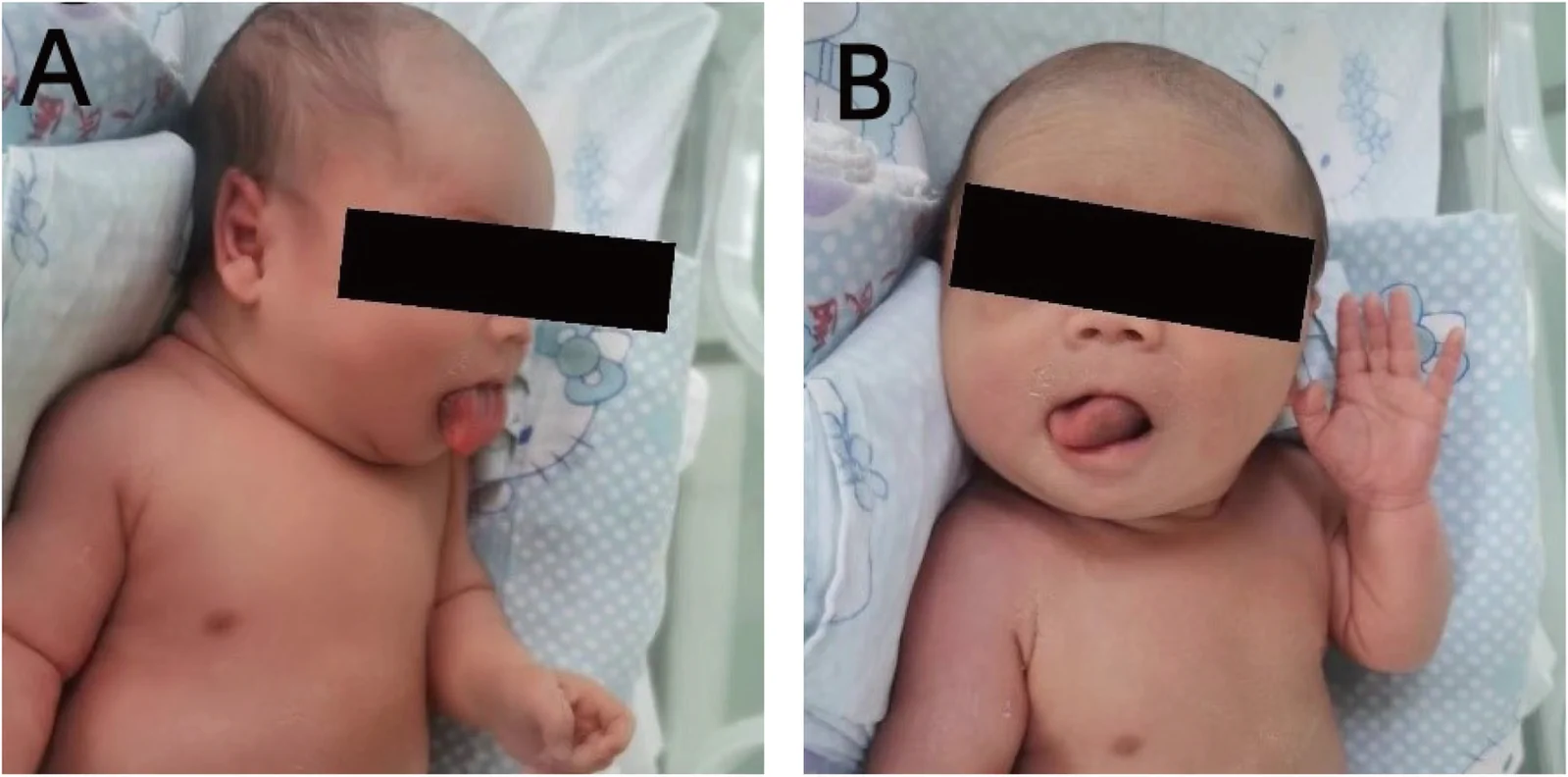
The condition of the child's tongue 6 to 10 days after birth. **(A,B)** represent the condition of the child's tongue at 6 days and 10 days after birth.

**Figure 4:**
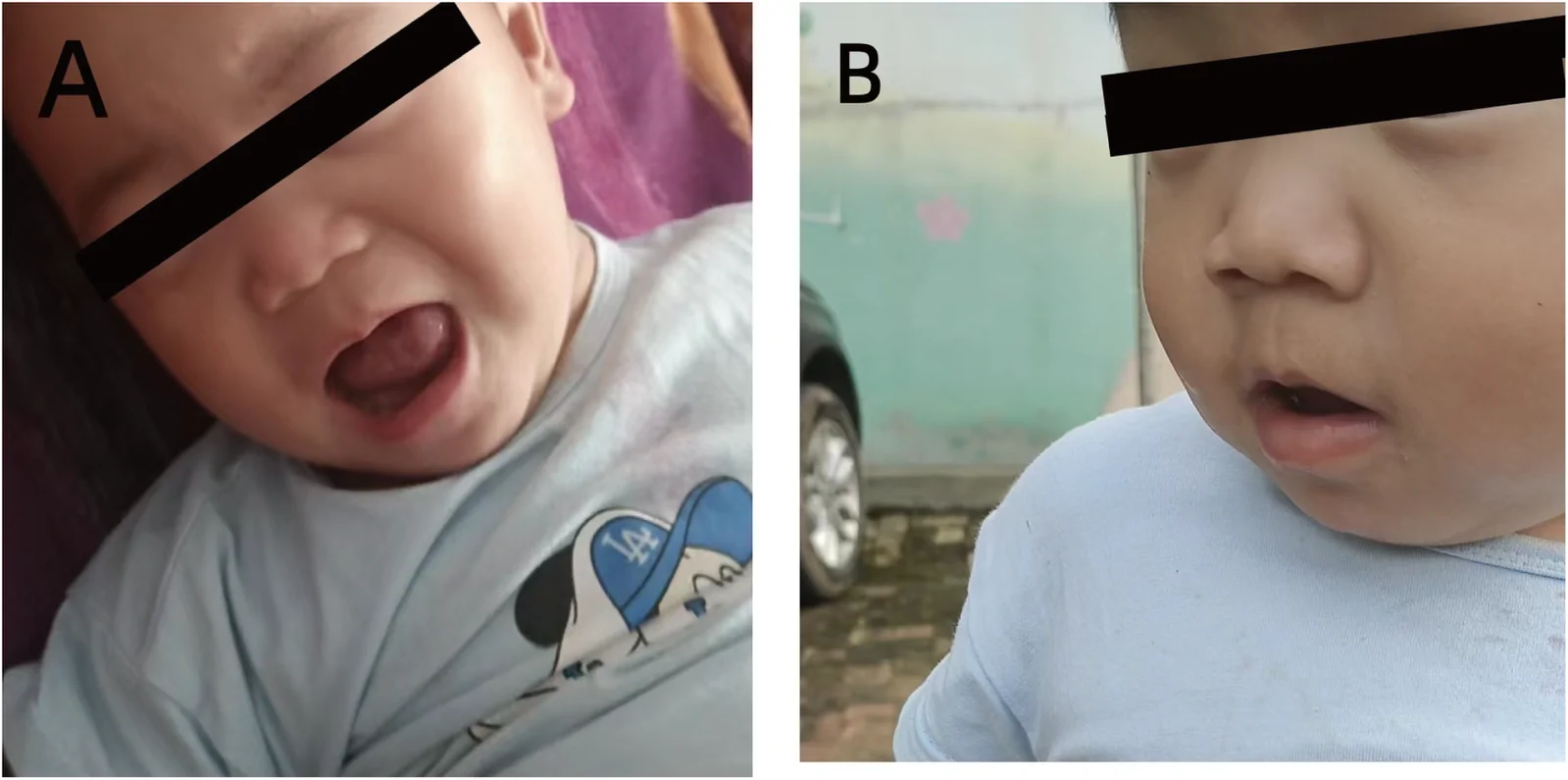
Monitoring the condition of the child's tongue in the later stage. **(A,B)** shown the condition of the child's tongue at the age of 2 years old.

The patient was discharged on day 15 of life in stable condition. She has been followed regularly for tumor surveillance with serial abdominal ultrasounds every 3–6 months, which have shown no evidence of Wilms tumor or hepatoblastoma. She underwent a partial glossectomy at 23 months of age with good cosmetic and functional results (Figures 3, 4). At her most recent follow-up at 2 years of age, her weight was 18 kg, height 91 cm, and her language, motor, and cognitive development were age-appropriate.

## Discussion

We report a case of BWS due to mosaic pUPD11 in a newborn with a classic clinical presentation but a normal prenatal CNV-seq result. This case offers several important lessons for clinicians regarding the diagnosis and management of the Beckwith-Wiedemann spectrum (BWSp).

First, and most critically, our case demonstrates that a normal prenatal low-coverage CNV-seq result does not rule out BWS, especially when the underlying molecular defect is a copy-number-neutral event such as mosaic pUPD. In this case, the CNV-seq was performed at ∼0.1–0.2 ×  depth with a ≥ 30% mosaicism reporting threshold, and the routine clinical pipeline did not implement AOH detection. Although the laboratory's bioinformatics platform is capable of AOH detection using SNP-based haplotype likelihood analysis combined with RNN at ultra-low depth (6), this analysis was not routinely performed for clinical reporting. Consequently, the 28.2 Mb AOH region was not identified. The patient's prenatal CNV-seq was interpreted as normal for clinically significant findings, which provided false reassurance. This is because the most common molecular causes of BWS are not CNVs but rather copy number-neutral events such as UPD and imprinting defects (7, 8). This case illustrates that the absence of a positive finding on routine CNV-seq does not exclude pUPD-related BWS, and targeted MS-MLPA testing is required when clinical suspicion exists (6). We acknowledge that a quantitative estimation of the mosaic level was not feasible from our WES data, as the B-allele frequency (BAF) plot and raw sequencing files were not accessible from the clinical diagnostic report. While BAF analysis from WES or SNP-array data can provide semi-quantitative estimates of mosaic UPD proportion by measuring the deviation from the expected 0.5 heterozygous allele ratio within the AOH region, this approach requires access to raw alignment files and is subject to sequencing depth limitations. Future studies with available WES raw data should consider BAF-based quantification to better characterize the genotype-phenotype correlation in mosaic pUPD. In our patient, the pUPD was initially suggested by a 28.2 Mb AOH region on whole-exome sequencing data, but definitive diagnosis required MS-MLPA, which is specifically designed to assess methylation status. Therefore, for any infant with clinical features suggestive of BWS (e.g., omphalocele + macroglossia + macrosomia), clinicians should proceed directly to MS-MLPA testing, regardless of prior normal array or karyotype results. In accordance with the 2018 consensus guidelines, a clinical score of 2 or more points should prompt direct MS-MLPA testing, and our case reinforces this recommendation. The prenatal CNV-seq was performed before BWS was clinically suspected and was not intended as a substitute for methylation testing.

Second, our report highlights the successful management of a giant omphalocele in a BWS patient through primary neonatal repair. Giant omphaloceles (defect >4 cm) are often managed with staged reduction or silo placement to avoid abdominal compartment syndrome. Our case, with a 4 cm defect containing most of the small intestine, colon, and part of the bladder, was successfully repaired in a single primary operation. This success was made possible by a multidisciplinary team approach involving obstetricians, neonatologists, pediatric surgeons, and anesthesiologists. Early primary repair allowed for prompt initiation of enteral nutrition and likely contributed to the patient's excellent growth and shortened hospital stay (discharged on day 15). We suggest that, with adequate neonatal intensive care and surgical expertise, primary repair is a feasible and preferable option even for giant omphaloceles in BWS patients.

Third, our patient's favorable outcome underscores the importance of long-term, risk-stratified tumor surveillance. Patients with pUPD, like our case, carry a cumulative childhood tumor risk of approximately 16%, primarily for Wilms tumor and hepatoblastoma (9–11). We followed the international consensus recommendation for pUPD patients, performing serial abdominal ultrasounds and serum alpha-fetoprotein (AFP) measurements every 3-4 months until at least 4 years of age, with continued renal ultrasound surveillance until age 7 (5, 9–11). At 2 years of follow-up, no tumors have been detected, and all AFP levels showed the expected age-appropriate decline. This supports the efficacy of regular surveillance in this high-risk population. We acknowledge that quantitative estimation of the mosaic proportion was not feasible, as our clinical MS-MLPA report provided only qualitative methylation patterns (ICR1 hypermethylation and ICR2 hypomethylation) without reporting methylation dosage ratios or methylation index (MI). Additionally, we cannot exclude tissue-specific mosaicism, since molecular testing was performed solely on peripheral blood and affected tissues were not sampled. As previously reported, mosaic pUPD levels may vary substantially between blood and other tissues, and blood-based testing may underestimate the true mosaic burden in BWS patients.

Finally, this case contributes to the growing understanding of the genotype-phenotype correlation in BWSp. Consistent with the literature (12, 13), our patient with pUPD presented with the triad of omphalocele, macroglossia, and macrosomia, without hyperinsulinism or hemihyperplasia. The successful management of her macroglossia with a single surgical procedure at 23 months is in line with reports that about 40% of BWS patients require surgical intervention for macroglossia, primarily to improve feeding, speech, and cosmesis.

In conclusion, this case provides a clear clinical reminder that BWS, particularly the pUPD subtype, can be missed by routine prenatal CNV-seq. A high index of clinical suspicion is paramount, and MS-MLPA remains the confirmatory diagnostic test of choice. With appropriate multidisciplinary management of omphalocele and macroglossia, along with diligent tumor surveillance, patients with BWS-pUPD can achieve excellent outcomes.

## Data Availability

The raw data supporting the conclusions of this article will be made available by the authors, without undue reservation.
